# Obesity survival paradox in pneumonia: a meta-analysis

**DOI:** 10.1186/1741-7015-12-61

**Published:** 2014-04-10

**Authors:** Wei Nie, Yi Zhang, Sun Ha Jee, Keum Ji Jung, Bing Li, Qingyu Xiu

**Affiliations:** 1 Department of Respiratory Medicine, Shanghai Changzheng Hospital, Second Military Medical University, 415 Fengyang Road, Shanghai 200003, China; 2 Department of Clinical Nutrition, The 452nd Hospital of PLA, Chengdu 610000, Sichuan, China; 3 Department of Epidemiology and Health Promotion, Graduate School of Public Health, Yonsei University, Seoul 120-749, Korea

**Keywords:** Body mass index, Obesity, Pneumonia, Dose–response relationship, Meta-analysis

## Abstract

**Background:**

It is unclear whether an ‘obesity survival paradox’ exists for pneumonia. Therefore, we conducted a meta-analysis to assess the associations between increased body mass index (BMI), pneumonia risk, and mortality risk.

**Methods:**

Cohort studies were identified from the PubMed and Embase databases. Summary relative risks (RRs) with their corresponding 95% confidence intervals (CIs) were calculated using a random effects model.

**Results:**

Thirteen cohort studies on pneumonia risk (n = 1,536,623), and ten cohort studies on mortality (n = 1,375,482) were included. Overweight and obese individuals were significantly associated with an increased risk of pneumonia (RR = 1.33, 95% CI 1.04 to 1.71, *P* = 0.02, *I*^*2*^ = 87%). In the dose–response analysis, the estimated summary RR of pneumonia per 5 kg/m^2^ increase in BMI was 1.04 (95% CI 1.01 to 1.07, *P* = 0.01, *I*^*2*^ = 84%). Inversely, overweight and obese subjects were significantly associated with reduced risk of pneumonia mortality (RR = 0.83, 95% CI 0.77 to 0.91, *P* < 0.01, *I*^*2*^ = 34%). The estimated summary RR of mortality per 5 kg/m^2^ increase in BMI was 0.95 (95% CI 0.93 to 0.98, *P* < 0.01, *I*^*2*^ = 77%).

**Conclusions:**

This meta-analysis suggests that an ‘obesity survival paradox’ exists for pneumonia. Because this meta-analysis is based on observational studies, more studies are required to confirm the results.

## Background

The prevalence of obesity has dramatically increased in the last two decades [1]. The diagnosis of obesity is often based on body mass index (BMI), calculated as weight in kilograms divided by height in meters squared (kg/m^2^). The ideal BMI is between 18.5 and 24.9. Being overweight is considered as having a BMI between 25 and 29.9, and being classified as obese falls into a BMI of 30.0 or greater [2]. Obesity is associated with an increased risk of cardiovascular disease and type 2 diabetes [3,4]. However, an inverse relationship between obesity and mortality has been described in patients with heart failure, coronary heart disease, and diabetes [5-7]. This phenomenon is known as the ‘obesity survival paradox.’

Pneumonia is one of the most common infectious diseases; however, there is uncertainty about the association between obesity and pneumonia risk or pneumonia mortality [8-28]. For example, Baik *et al.*[9] suggested that obesity was directly associated with the development of community-acquired pneumonia (CAP). However, Phung *et al.*[19] did not find that obesity was significantly associated with pneumonia risk. Takata *et al.*[24] indicated that mortality risk was not different between obese pneumonia patients and normal weight patients. However, other studies reported that obese subjects with pneumonia had lower mortality compared to normal weight subjects [26-28]. Thus, whether the ‘obesity survival paradox’ exists in pneumonia is still unclear.

To date, no meta-analysis has shown whether an ‘obesity survival paradox’ exists for pneumonia. The aim of this meta-analysis was to investigate the relationships between elevated BMI, pneumonia risk, and mortality.

## Methods

This meta-analysis was performed according to a predetermined protocol described in the following paragraphs, using standard systematic review techniques, as outlined by the Meta-Analysis of Observational Studies in Epidemiology (MOOSE) criteria [29].

### Literature search

A literature search was performed (WN and YZ) using the PubMed search engine, with the database being last accessed on 15 June 2013. The Embase database was also searched for relevant studies published up to June 2013. References from relevant articles were manually checked for further studies. The detailed search strategy is presented in the Additional file 1.

### Study selection

Two reviewers (WN and YZ) independently screened the abstracts of papers identified by the literature search, retrieved potentially relevant studies and determined study eligibility. Studies were included if: (1) the study design was a prospective or retrospective cohort study; (2) the exposure of interest was BMI; (3) they reported adjusted relative risks (RRs), hazard ratios (HRs) or odds ratios (ORs) with corresponding 95% confidence intervals (CIs), or provided a RR/HR/OR with corresponding 95% CI per unit increment in BMI; and (4) the outcome was pneumonia incidence or mortality. If the same cohort was used in more than one publication, we included the publication that reported the results in greater detail or, if similar, the one with the largest number of cases. Data published only in abstract form were excluded. Case reports, review articles and commentary articles were also excluded. Studies with pediatric participants or pregnant populations were not included.

### Data collection and methodological quality assessment

From each study, two reviewers (WN and YZ) independently extracted the first author, publication year, study design, location where the study was performed, number of cases and cohort size, gender and age of study participants, follow-up duration, method for assessing height and weight, ascertainment of pneumonia, type of pneumonia, BMI category, adjusted RR/HR/OR and the corresponding 95% CI, and covariates controlled for multivariable analysis. The authors of the relevant studies were contacted by Email if more information was needed.

Two independent reviewers (WN and YZ) completed the quality assessment. The Newcastle–Ottawa Scale (NOS) was used to evaluate the methodological quality, which scored studies by the selection of the study groups, the comparability of the groups and the ascertainment of the outcome of interest [30]. Discrepancies were resolved by consensus and discussion. The detailed criteria of the methodological quality assessment are in Additional file 2.

### Statistical analysis

For pneumonia risk and pneumonia mortality risk, we calculated summary RRs and 95% CIs for overweight and obesity *versus* normal weight. The random effects model was utilized. HRs and ORs were regarded as equivalent to RRs in cohort studies. If a study reported results specifically for men and women, respectively, we combined the sex-specific RR estimates using a fixed-effects model before combining with other studies.

In dose–response analysis, we calculated the RR per 5-unit increase in the BMI levels for each study. The average of the natural logarithm of the RRs was estimated and the RR from each study was weighted by the inverse of its variance. A two-tailed *P* < 0.05 was considered statistically significant. We also combined the sex-specific estimates using a fixed-effects model to generate an estimate for both genders combined. The method described by Greenland and Longnecker [31] was used for the dose–response analysis and study-specific slopes (linear trends) and 95% CIs were computed from the natural logs of the RRs and CIs across categories of BMI. This method requires the distribution of case and person-years and the median level of BMI in each category to the corresponding RR for each study (the RRs with estimates for at least three quantitative exposure categories are known). The midpoint between the upper and lower boundary for each BMI category was assigned to the corresponding RR estimate. For studies with an open-ended highest or lowest BMI category, we assumed that the amplitude was the same as the closest adjacent category. Random effects models were used to pool the respective results. The dose–response results in the forest plot were presented for a 5 kg/m^2^ BMI increment.

Nonlinear dose–response curves were plotted using restricted cubic splines for each study, using knots fixed at percentiles 10%, 50% and 90% through the distribution; then these were combined using multivariate meta-analysis [32-34].

Statistical heterogeneity among studies was evaluated using the Q and *I*^*2*^ statistics. For the *I*^*2*^ metric, we considered low, moderate and high *I*^*2*^ values to be 25%, 50% and 75%, respectively. We examined the role of several potential sources of heterogeneity by subgroup analyses according to study design, gender, ascertainment of case, pneumonia type, assessment of anthropometry, and duration of follow-up. Meta-regression was also performed to find the sources of heterogeneity. Sensitivity analysis was conducted by excluding one study at a time to explore whether the results were driven by one large study or by a study with an extreme result. Potential small study effects, such as publication bias, were investigated with funnel plots.

All statistical analyses were performed with the Stata software (version 12.0, Stata Corporation, College Station, Texas). A threshold of *P* < 0.1 was used to decide whether heterogeneity was present. In other cases, *P* values were two sided with a significance level of 0.05.

## Results

### Literature search

**Figure 1:**
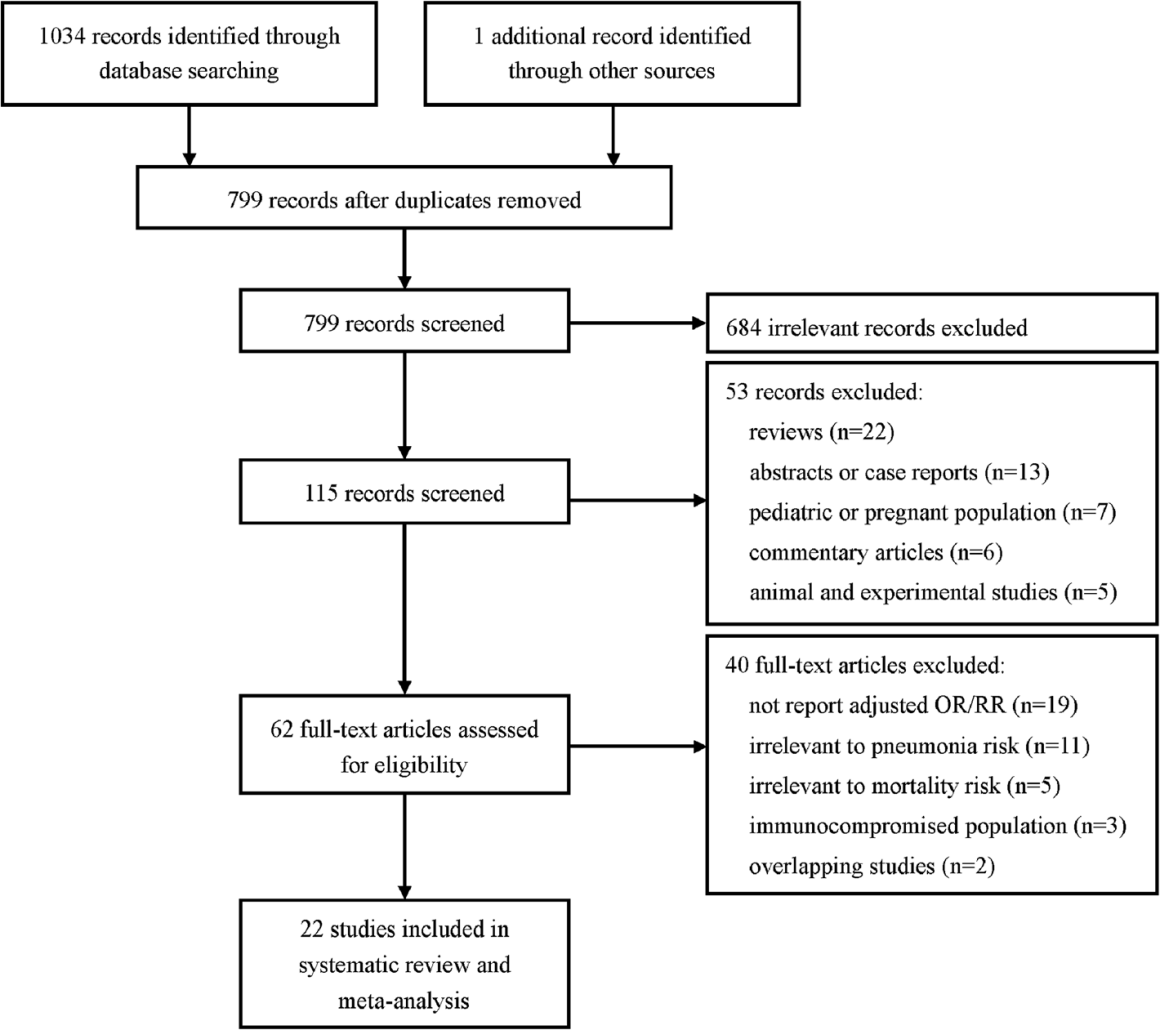
Flow of study identification, inclusion and exclusion.

The process of identifying relevant studies is shown in Figure 1. The initial search produced 1,035 studies from the PubMed and Embase databases. After exclusion of duplicates and irrelevant studies, 115 potentially eligible studies were selected. After detailed evaluations, 21 studies were selected for final meta-analysis [8-28]. A manual search of reference lists from these studies did not yield any new eligible study. Several studies investigated the association between BMI and mortality. We contacted these authors to get additional data on pneumonia mortality. Dr. Sun Ha Jee and colleague shared their data [35]. Finally, 22 studies were included in this meta-analysis [8-28,35].

### Study characteristics

Twelve cohort studies (n = 1,536,623) investigated the association between BMI and pneumonia risk [8-19], and ten studies (n = 1,375,482) assessed the association between BMI and pneumonia mortality [20-28,35]. There were seven retrospective cohort studies [11,15-18,25,26] and fifteen prospective cohort studies [8-10,12-14,19-24,27,28,35]. The durations of follow-up varied from 1 year to 15.8 years. Sixteen studies collected measured BMI [8,10-15,19,20,23-28,35]; three studies collected self-reported data [9,17,21]. The characteristics of each study are presented in Table 1. The methodological quality assessment is provided in Additional file 2. The Preferred Reporting Items for Systematic Reviews and Meta-Analyses (PRISMA) checklist for meta-analysis is provided in Additional file 3.

**Table 1:** Characteristics of included cohort studies

**Author**	**Study design**	**Year**	**Location**	**Gender**	**Age (y)**	**Years of follow-up (y)**	**Assessment of weight and height**	**Sample size**	**Cases ascertainment**	**No. of cases**	**Type**	**BMI category (kg/m**^**2**^**)**	**Adjustment for covariates**
**Incidence**													
Delgado-Rodriguez	PC	1997	Spain	Mixed	53	2	Measured	1,483	Physician-diagnosed	19	HAP	<33.75, ≥33.75	Mechanical ventilation, upper abdominal surgery, COPD, NNIS index
Baik	PC	2000	USA	Mixed	56.3 (men) 36.4 (women)	6	Self-reported	104,491	Physician-diagnosed	595	CAP	<21, 21–22.9, 23–24.9, 25–26.9, 27–29.9, ≥30	Age, smoking status, physical activity, alcohol intake
Newell	PC	2007	USA	Mixed	45	5	Measured	1,543	NA	315	HAP	18.5–24.9, 25.0–29.9, 30.0–39.9, ≥40	Age, gender, injury severity score, revised trauma score
Yap	RC	2007	Australia	Mixed	66.4	6	Measured	3,968	Physician-diagnosed	174	HAP	<20, 20–30, 30–40, ≥40	Age, gender, diabetes, hypercholesterolemia, renal impairment, preoperative dialysis, hypertension, cerebrovascular disease, peripheral vascular disease, lung disease, NYHA class IV, severe LV impairment, mean PA pressure, emergency status and total bypass time
Dossett	PC	2008	USA	Mixed	45	NA	Measured	1,406	Physician-diagnosed	446	HAP	<18.5, 18.5–24.9, 25.0–29.9, 30.0–39.9, ≥40	Age, gender, TRISS, AIS head score
Mannino	PC	2008	USA	Mixed	>45	3	Measured	20,375	ICD-9 codes 480-486	214	CAP	<20, 20–24, 25–29, ≥30	Age, gender, race, smoking status, education level, diabetes status, cardiovascular disease status
Kornum	PC	2010	Denmark	Mixed	50-64	11.8	Measured	48,551	ICD-10 codes J12.x–J18.x, ICD-10codes A709.x, ICD-10 codes A481.x	2,112	CAP	<22.5, 22.5–24.9, 25.0–29.9,30.0–34.9, ≥35	Age, smoking status, alcohol intake, schooling, educational level
Morgan	RC	2010	USA	Mixed	>20	1	Measured	196,684	Physician-diagnosed	134	CAP	<18.5, 18.5–24.9, 25.0–29.9, 30-39.9, ≥40	Age, gender, chronic disease
Blumentals	RC	2011	UK	Mixed	>18	7	NA	1,074,315	NA	877	CAP	<18.5, 18.5–24.9, 25.0–29.9, ≥30	Age, gender, type 2 diabetes, hypertension, statin use, antibiotic use, cigarette smoking status, influenza vaccination status, and year of index date
Kwong	RC	2011	Canada	Mixed	>18	12	Self-reported	82,545	ICD-9 codes 480–486, ICD-10-CM codes J10-J18	228	CAP	<18.5, 18.5–24.9, 25.0–29.9, ≥30	Age, gender, influenza vaccination status, rural residence, income quintile, smoking status, previous hospitalizations, previous outpatient visits, and the presence of chronic disease
Viasus	RC	2011	Spain	Mixed	39	NA	NA	585	Physician-diagnosed	11	CAP	<30, 30–39.9, ≥40	Age, gender, smoking, alcohol drinking, comorbid condition, influenza vaccine, early antiviral treatment, concomitant and/or secondary bacterial co-infection
Phung	PC	2013	Australia	Mixed	33	15.6	Measured	677	ICD-9 codes 480–486, ICD-10 codes J12-J18	141	CAP	<18.5, 18.5–24.9, 25.0–29.9, ≥30	Age, smoking, alcohol consumption status
**Mortality**													
LaCROIX	PC	1989	USA	Mixed	65.8	12	Measured	5474	ICD-9 codes 480-486	76	CAP	Lowest quartile, Second quartile,Third quartile, Highest quartile	Age
Salive	PC	1993	USA	Mixed	74	6	Self-reported	10,269	ICD-9 codes 480-486	403	CAP	Lowest quartile, Second quartile,Third quartile, Highest quartile	Age, race, education, co-morbidities, smoking, peak expiratory flow, emphysema, exercise
Lange	PC	1995	Denmark	Mixed	30-70	12	NA	13,423	ICD-8 codes 480-486	260	CAP	<20, 20–29, ≥30	Age, predicted forced expiratory volume in one second
Jee	PC	2006	Korea	Mixed	46	15	Measured	1,213,829	ICD-10	962	CAP	<18.5, 18.5–19.9, 20.0–21.4, 21.5–22.9, 23.0–24.9, 25.0–26.4,26.5–27.9, 28.0–29.9, 30.0–31.9	Age, smoking status, alcohol intake, physical exercise, physical activity
Inoue	PC	2007	Japan	Mixed	57.6	13	Measured	110,792	ICD-9 codes 480-486 ICD-10 codes J12-J18	1,082	CAP	10.0–17.9, 18.0–22.9, 23.0–24.9,25.0–32.9	Age, diabetes mellitus
Takata	PC	2007	Japan	Mixed	80	4	Measured	697	ICD-10	19	CAP	<18.5, 18.5–24.9, ≥25	Gender, tobacco use, alcohol use, weight loss, current outpatient, systolic blood pressure, physical activity, functional status, marital status, and levels of total serum cholesterol and glucose
Corrales-Medina	RC	2011	USA	Mixed	65.5	7	Measured	266	Physician-diagnosed	31	CAP	<18.5, 18.5–24.9, 25.0–29.9, >30	Age, race, cancer, CURB-65
King	RC	2012	USA	Mixed	67.5	7	Measured	18,746	ICD-9 codes 480-487	3,340	CAP	<18.5, 18.5–24.9, 25.0–29.9, 30-39.9, >40	Age, gender, marital status, race, count of current medications, medical and psychiatric comorbid conditions, alcohol abuse, tobacco use, and drug abuse
Kahlon	PC	2012	Canada	Mixed	68	2	Measured	907	Physician-diagnosed	79	CAP	<18.5, 18.5–24.9, 25.0–29.9, >30	Age, functional status, prior pneumococcal vaccination, chest radiograph confirmation, PSI
Singanayagam	PC	2013	UK	Mixed	50-78	NA	Measured	1,079	Physician-diagnosed	103	CAP	<18.5, 18.5–24.9, 25.0–29.9,30.0–34.9	Age, gender, co-morbidities, current smoking, functional status, nursing home residence, PSI

AIS, abbreviated injury score; BMI, body mass index; CAP, community-acquired pneumonia; COPD, chronic obstructive pulmonary diseases, CURB-65, confusion, urea >7 mmol/L, respiratory rate ≥30 breaths/minute, low blood pressure and age ≥65 years; F, female; HAP, hospital-acquired pneumonia; ICD, International Classification of Diseases; LV, left ventricle; M, male; NA, not available; NNIS, National Nosocomial Infection Surveillance; NYHA, New York Heart Association; PC, prospective cohort; PSI, Pneumonia Severity Index; RC, retrospective cohort; TRISS, trauma-related injury severity score.

### Quantitative data synthesis

#### Pneumonia risk (overweight and obesity versus normal weight)

**Figure 2:**
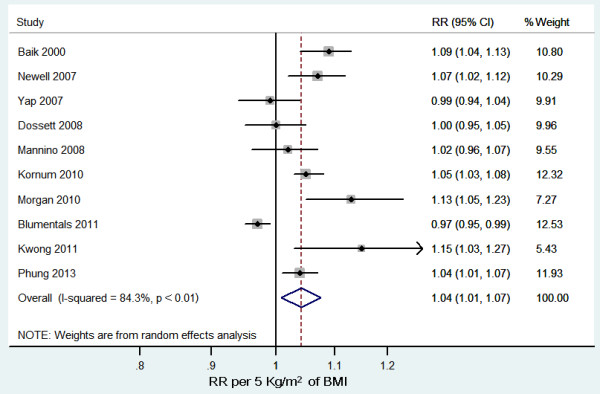
**Relative risks of pneumonia risk per 5 kg/m2 increase in body mass index.** CI: indicates confidence interval; and RR: risk ratio.

**Figure 3:**
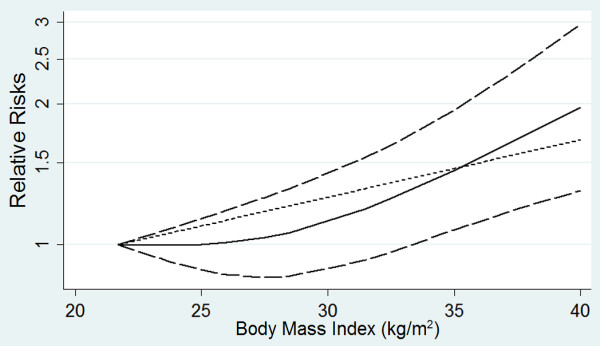
**Dose–response relationship between body mass index and relative risk of pneumonia.** Body mass index was modeled with a nonlinear trend (black continuous line) in a random effects meta-regression model. Long-dashed black lines represent 95% confidence intervals. Short-dashed black lines represent the linear trend. The vertical axes are on a log scale.

Compared with normal weight individuals, overweight and obese individuals were associated with a significantly increased risk of pneumonia (RR = 1.33, 95% CI 1.04 to 1.71, *P* = 0.02, *I*^*2*^ = 87%). A small-study effect was demonstrated using a funnel plot [see Additional file 4]. Ten studies reported RRs for categorized BMI levels [9-17,19]. Thus, we included these studies for the dose–response analysis. The summary RR was 1.04 (95% CI 1.01 to 1.07, *P* = 0.01, *I*^*2*^ = 84%; Figure 2). A potentially nonlinear dose–response relationship was not detected (*P* > 0.05; Figure 3). We found evidence of a small-study effect as assessed by funnel plot [see Additional file 5].

We conducted a sensitivity analysis by omitting one study at a time and calculating the pooled RRs for the remainder of the studies. This sensitivity analysis showed that the results were not changed (data not shown). The potential sources of heterogeneity were explored by stratifying analyses. Studies comparing overweight and obese subjects with normal weight subjects, and studies assessing dose–response associations were explored, respectively. The positive relationship between BMI and risk of pneumonia was significant in subgroups by ascertainment of case and assessment of weight and height [see Additional file 6]. However, in subgroups of retrospective cohort studies, male population, female population, hospital-acquired pneumonia, and longer follow-up duration, the positive relationships were not statistically significant [see Additional file 6]. A meta-regression found that assessment of weight and height might be the source of the high heterogeneity.

#### Pneumonia mortality risk (overweight and obese versus normal weight)

Overweight and obese individuals were associated with decreased mortality risk (RR = 0.83, 95% CI 0.77 to 0.91, *P* < 0.01, *I*^*2*^ = 34%). A small-study effect was revealed by the funnel plot [see Additional file 7].

**Figure 4:**
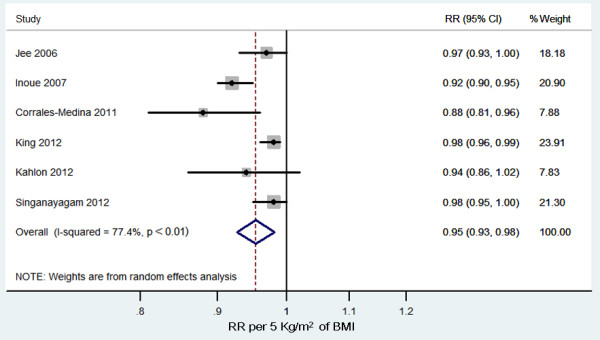
**Relative risks of pneumonia mortality risk per 5 kg/m**^**2**^**increase in body mass index.** CI: indicates confidence interval; and RR: risk ratio.

**Figure 5:**
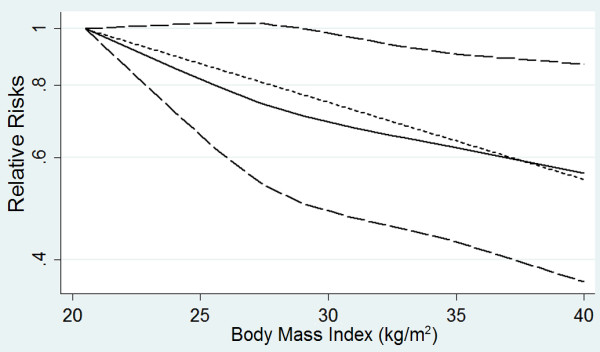
**Dose–response relationship between body mass index and relative risk of pneumonia mortality.** Body mass index was modeled with a nonlinear trend (black continuous line) in a random effects meta-regression model. Long-dashed black lines represent 95% confidence intervals. Short-dashed black lines represent the linear trend. The vertical axes are on a log scale.

Six cohort studies were identified in the dose–response analysis [23,25-28,35]. The summary RR was 0.95 (95% CI 0.93 to 0.98, *P* < 0.01, *I*^*2*^ = 77%; Figure 4). There was no evidence of a nonlinear relationship with BMI (*P* = 0.44; Figure 5). The shape of the funnel plot was asymmetrical, suggesting that there was a small-study effect [see Additional file 8].

In sensitivity analyses, no result was significantly altered when excluding studies one by one (data not shown). Stratified analyses were defined by study design, gender, assessment of weight and height, and follow-up duration. Studies comparing overweight and obese subjects with normal weight subjects, and studies assessing dose–response associations were explored, respectively. The inverse relationship between BMI and risk of mortality was significant in subgroups that were defined by ascertainment of case and study design. However, in the female subgroup or studies with shorter follow-up duration, the inverse relationship was not statistically significant [see Additional file 9]. Study design was found to be the major source of heterogeneity by meta-regression.

## Discussion

In this meta-analysis, we showed that an ‘obesity survival paradox’ might exist for pneumonia. On the one hand, there was a positive association of obesity with pneumonia risk. On the other hand, pneumonia mortality was lower for patients with high BMI compared to normal BMI.

In a recent meta-analysis, Phung and coworkers found a J-shaped relationship between BMI and risk of CAP and a U-shaped relationship between BMI and risk of influenza-related pneumonia [36]. Our results were partly similar to this previous report. In the current meta-analysis, we only investigated the association between higher BMI and pneumonia risk, but did not assess the association between subnormal BMI and pneumonia risk. Thus, the shape of our dose–response relationship deviated from J-shaped or U-shaped.

There were several potential explanations for why obese individuals may have higher risk of pneumonia. First, obesity is often accompanied by co-morbid conditions, such as gastroesophageal reflux disease [37]. When reflux of gastric fluid occurs, the fluid can be aspirated into the respiratory tract resulting in pneumonia [37]. Second, obesity has been shown to be an independent predictor of diabetes and asthma. These two diseases are also important risk factors for pneumonia [38,39]. Third, a recent study reported that a higher BMI led to lower 25(OH)D_3_ levels [40]. More recently, Aregbesola and colleagues found that subjects with a lower serum 25(OH)D_3_ concentration had a higher risk of pneumonia [41]. Fourth, leptin (*ob/ob*) and leptin receptor (*db/db*) deficient mice showed severe immune abnormalities and greater susceptibility to viral and bacterial infection [42]. Individuals with this leptin defect also exhibited greater susceptibility to respiratory infections [43]. Therefore, leptin plays an important role in the human immune response to infectious disease. Although there were elevated leptin levels in obese subjects, leptin resistance often coexisted with these persons [44]. Taken together, these results suggest that individuals with high BMI might have an increased risk of pneumonia compared to subjects with normal BMI.

This present meta-analysis suggested a survival advantage for obese patients with pneumonia. A study by LaCroix *et al.* showed that the risk of pneumonia mortality was 2.6 times higher in men of the lowest BMI quartile compared to the highest quartile [20]. Another study by Salive *et al*. indicated that the highest two quartiles of BMI had a significantly reduced risk of mortality compared with the lowest quartile [21]. We propose three explanations for the inverse relationship between obesity and the risk of pneumonia mortality. First, obese individuals have a higher risk of developing coronary heart disease, type II diabetes, and heart failure [45]. Thus, obese patients with pneumonia may receive optimal medical treatment or aggressive treatment. This may lead to a reduction in mortality. Second, tumor necrosis factor-alpha (TNF-α) is a potential proinflammatory cytokine that plays a critical role in inflammatory and immune responses. Puren *et al.*[46] indicated that the plasma level of TNF-α is a marker of pneumonia severity. Adipose tissue is known to produce soluble TNF-α receptors [47]. Additionally, recent studies showed that obese patients with pneumonia had lower pneumonia severity index scores and plasma levels of C-reactive protein [27,28]. Third, as with other diseases, patients with pneumonia who are at a normal weight may not have enough metabolic reserve to counteract the increased catabolic stress. These patients may be particularly vulnerable to the adverse pathophysiologic consequences of a limited metabolic reserve.

There are several limitations of the current study. First, a meta-analysis of observational studies inherits the limitation of the original studies. Although most studies adjusted for potential confounders, such as age, gender, smoking and underlying diseases, the possibility of residual confounding cannot be ruled out. Because this meta-analysis investigated only BMI, we cannot exclude the possibility that the observed associations may be confounded by other lifestyle factors, such as lower physical activity or dietary factors. Second, the number of available studies that were included in this meta-analysis was moderate. Therefore, the results could be influenced by some factors,such as random error. Third, most of the studies used International Classification of Diseases (ICD)-9 or ICD-10 codes to classify pneumonia. van de Garde and coworkers suggested that ICD-9 codes showed modest sensitivity for detecting CAP, leaving at least one quarter of pneumonia cases undetected [48]. Fourth, statistical heterogeneity was detected when quantitative pooling was performed. In addition, small-study effects were detected in this study. Thus, caution with interpretation of the results is necessary, and these results should be confirmed by future studies.

## Conclusions

This meta-analysis suggests that obese individuals may be at higher risk for pneumonia, but they might have a lower mortality risk. Additional prospective studies with adjustment for more confounding factors are warranted before a conclusion can be drawn.

## Abbreviations

BMI: body mass index; CAP: community-acquired pneumonia; CI: confidence interval; HAP: hospital-acquired pneumonia; ICD: International Classification of Diseases; NOS: Newcastle–Ottawa Scale; RR: risk ratio; TNF-α: tumor necrosis factor-alpha.

## Competing interests

The authors declare that they have no competing interests.

## Authors’ contributions

WN searched the papers, created tables, and wrote the manuscript. YZ searched the papers, created tables, and wrote the manuscript. SHJ analyzed and provided data. KJJ analyzed and provided data. BL reviewed the study proposal and contributed to the manuscript writing. QX reviewed the study proposal and contributed to the manuscript writing. All authors read and approved the final manuscript.

## Pre-publication history

The pre-publication history for this paper can be accessed here:

http://www.biomedcentral.com/1741-7015/12/61/prepub

## Supplementary Material

Additional file 1 Search strategy. Click here for file

Additional file 2 Check list for methodological quality assessment and quality scores of cohort studies using the Newcastle-Ottawa Scale. Click here for file

Additional file 3 PRISMA checklist. Click here for file

Additional file 4 Funnel plot of the association between obesity and pneumonia risk. Click here for file

Additional file 5 Funnel plot of the association between obesity and pneumonia risk in dose-response analysis. Click here for file

Additional file 6 Subgroup analyses of pneumonia risk, overweight and obesity versus normal weight and dose–response analyses, respectively. Click here for file

Additional file 7 Funnel plot of the association between obesity and pneumonia mortality risk. Click here for file

Additional file 8 Funnel plot of the association between obesity and pneumonia mortality risk in dose-response analysis. Click here for file

Additional file 9 Subgroup analyses of pneumonia mortality risk, overweight and obesity versus normal weight and dose–response analyses, respectively. Click here for file
