# Supplementary material for: Obesity survival paradox in pneumonia: a meta-analysis
Source: BMC Med. 2014 Apr 10;12:61. doi: 10.1186/1741-7015-12-61 (PMC4021571; doi:10.1186/1741-7015-12-61)
Supplement: Additional file 1 — Search strategy. [file 1741-7015-12-61-S1.pdf]

PubMed

1. Obesity[MeSH]

2. obesity[TIAB] OR obese[TIAB]

3. Adiposity[MeSH]

4. adiposity[TIAB] OR adipose[TIAB] OR adipos*[TIAB]

5. body size [TIAB]

6. Body Size[MeSH]

7. Body Mass Index[MeSH]

8. body mass index [TIAB] OR BMI [TIAB] OR body mass[TIAB]

9. 1 OR 2 OR 3 OR 4 OR 5 OR 6 OR 7 OR 8

10. Pneumonia[MeSH]

11. pneumoniae[TIAB] OR pneumon*[TIAB]

12. pneumococcal infections [TIAB] OR pneumococcal[TIAB] OR CAP[TIAB] OR community-acquired pneumonia [TIAB] OR HAP[TIAB] OR hospital-acquired pneumonia [TIAB]

13. 10 OR 11 OR 12

14. observational study [PT]

15. observational study [MeSH] OR observational study [TIAB]

16. longitudinal studies [MeSH] OR longitudinal studies [TIAB]

17. retrospective studies [MeSH] OR retrospective studies [TIAB]

18. cohort[TIAB] OR follow-up[TIAB] OR prospective cohort [TIAB] OR historical cohort [TIAB]

19. 14 OR 15 OR 16 OR 17 OR 18

20. 9 AND 13 AND 19

21. mortality[MeSH] OR mortality[TIAB]

22. death[MeSH] OR death* [TIAB]

23. survive[TIAB] OR surviv*[TIAB]

24. prognosis[TIAB] OR progno*[TIAB]

25. 21 OR 22 OR 23 OR 24

26. 20 AND 25

EMBASE

1. Obesity.mp. OR obesity/exp

2. adiposity:ab,ti OR adipose:ab,ti OR adipos*:ab,ti OR body size:ab,ti

3. BMI.mp. OR Body Mass Index.mp. OR body mass/exp

4. 1 OR 2 OR 3

5. pneumonia/exp

6. pneumon*:ab,ti OR pneumon*.mp. OR pneumonia.mp.

7. CAP:ab,ti OR HAP:ab,ti OR community-acquired pneumonia:ab,ti OR hospital-acquired pneumonia:ab,ti

8. 5 OR 6 OR 7

9. Cohort Studies/exp

10. epidemiology/

11. cohort$.tw.

12. observational study/exp OR longitudinal studies/exp OR retrospective studies/exp OR follow-up/exp OR prospective cohort/exp OR historical cohort/exp

13. 9 OR 10 OR 11 OR 12

14. 4 AND 8 AND 13

15. mortality/exp OR mortality.mp. OR mortality:ab,ti

16. death/exp OR death.mp. OR death*:ab,ti

17. survive.mp. OR survival/exp OR surviv*:ab,ti

18. prognosis/exp OR prognosis.mp. OR progno*:ab,ti

19. 15 OR 16 OR 17 OR 18

20. 14 AND 19
