# Supplementary material for: Obesity survival paradox in pneumonia: a meta-analysis
Source: BMC Med. 2014 Apr 10;12:61. doi: 10.1186/1741-7015-12-61 (PMC4021571; doi:10.1186/1741-7015-12-61)
Supplement: Additional file 2 — Check list for methodological quality assessment and quality scores of cohort studies using the Newcastle-Ottawa Scale. [file 1741-7015-12-61-S2.pdf]

Table S1. Check list for methodological quality assessment.

Selection

1) Representativeness of the exposed cohort

a) truly representative of the population in the community (if yes, one point)

b) somewhat representative of the population in the community (if yes, one point)

c) selected group of users eg nurses, volunteers (if yes, zero point)

d) no description of the derivation of the cohort (if yes, zero point)

2) Selection of the non exposed cohort

a) drawn from the same community as the exposed cohort (if yes, one point)

b) drawn from a different source (if yes, zero point)

c) no description of the derivation of the non exposed cohort (if yes, zero point)

3) Ascertainment of exposure

a) measured (if yes, one point)

b) self report (if yes, zero point)

c) no description (if yes, zero point)

4) Demonstration that outcome of interest was not present at start of study

a) yes (if yes, one point)

b) no (if yes, zero point)

Comparability

Comparability of cohorts on the basis of the design or analysis

a) study investigating pneumonia risk controls for age, gender, and smoke (if yes, two points; one point was assigned if one of these characteristics was not reported and other characteristics had been controlled for; no point was assigned if none of these characteristics had been controlled for)

6) study investigating pneumonia mortality controls for age, gender, and underling diseases (if yes, two points; one point was assigned if one of these characteristics was not reported and other characteristics had been controlled for; no point was assigned if none of these characteristics had been controlled for)

Outcome

1) Assessment of outcome

a) physician-diagnosed (if yes, one point)

b) record linkage (if yes, one point)

c) self report (if yes, zero point)

d) no description (if yes, zero point)

2) Was follow-up long enough for outcomes to occur

a) an adequate follow up period for outcome of interest (one point if follow-up duration>5 years)

b) no adequate follow up period (zero point if follow-up duration≤5 years)

3) Adequacy of follow-up rate of cohorts

a) adequacy of follow-up rate (one point if follow-up>80%)

b) no adequate follow up rate (zero point if follow-up≤80%)

Table S2. Quality scores of cohort studies using Newcastle-Ottawa Scale.

	Selection				Comparability	Outcome		
Study	Representativeness of the exposed cohort	Selection of the non exposed cohort	Ascertainment of exposure	Demonstration that outcome of interest was not present at start of study	Comparability of cohorts on the basis of the design or analysis	Assessment of outcome	Was follow-up long enough for outcomes to occur (>5 years)	Adequacy of follow-up rate of cohorts (>80%)
Delgado-Rodriguez	1	1	1	1	1	1	0	1
Baik	1	1	0	1	1	1	1	1
Newell	1	1	1	1	1	0	1	1
Yap	1	1	1	1	2	1	1	1
Dossett	1	1	1	1	1	1	0	1
Mannino	1	1	1	1	2	1	0	1
Kornum	1	1	1	1	2	1	1	1
Morgan	1	1	1	1	1	1	0	1
Blumentals	1	1	0	1	2	0	1	1
Kwong	1	1	0	1	2	1	1	1
Viasus	1	1	0	1	2	1	0	1
Phung	1	1	1	1	2	1	1	1
LaCROIX	1	1	1	1	1	1	1	1
Salive	1	1	0	1	2	1	1	1
Lange	1	1	0	1	1	1	1	1
Jee	1	1	1	1	1	1	1	1
Inoue	1	1	1	1	2	1	1	1

Takata	1	1	1	1	1	1	0	1
Corrales-Medina	1	1	1	1	1	1	1	1
King	1	1	1	1	2	1	1	1
Kahlon	1	1	1	1	1	1	0	1
Singanayagam	1	1	1	1	2	1	0	1
