# Supplementary figures and images for: Obesity survival paradox in pneumonia: a meta-analysis
Source: BMC Med. 2014 Apr 10;12:61. doi: 10.1186/1741-7015-12-61 (PMC4021571; doi:10.1186/1741-7015-12-61)

## Slide 1
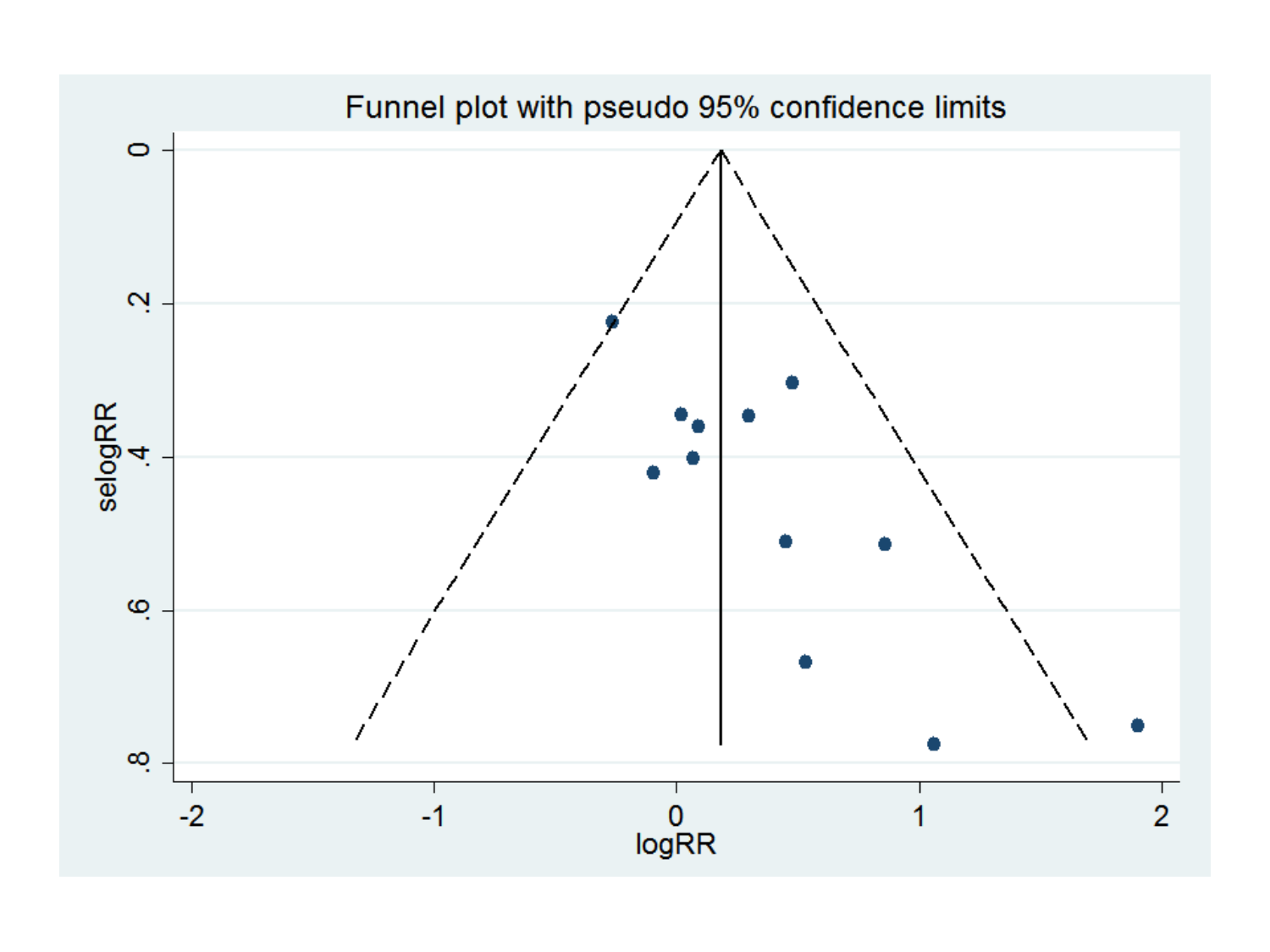

#

Supplement: Additional file 4 — Funnel plot of the association between obesity and pneumonia risk. [file 1741-7015-12-61-S4.pptx]

## Slide 1
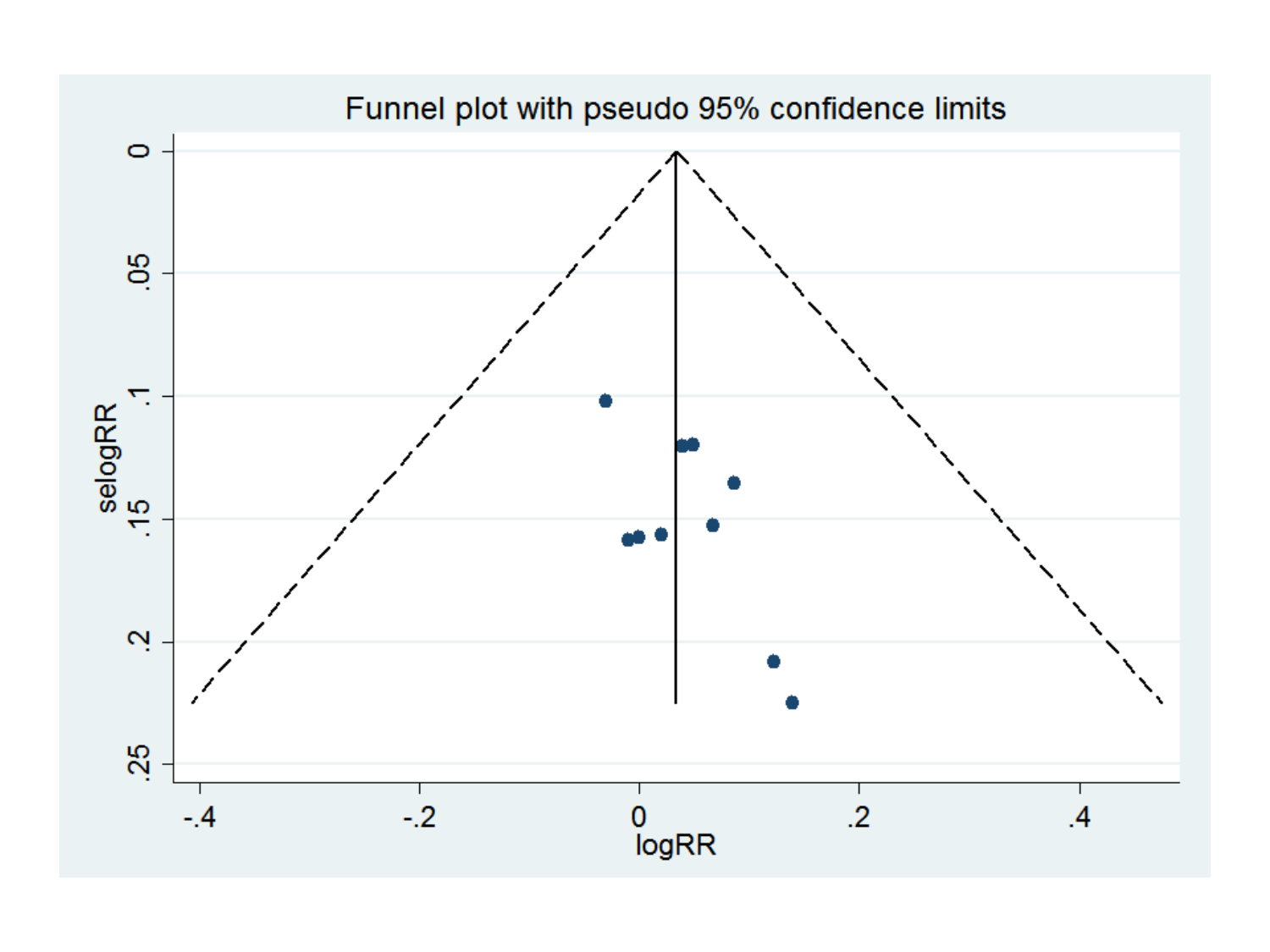

#

Supplement: Additional file 5 — Funnel plot of the association between obesity and pneumonia risk in dose-response analysis. [file 1741-7015-12-61-S5.pptx]

## Slide 1
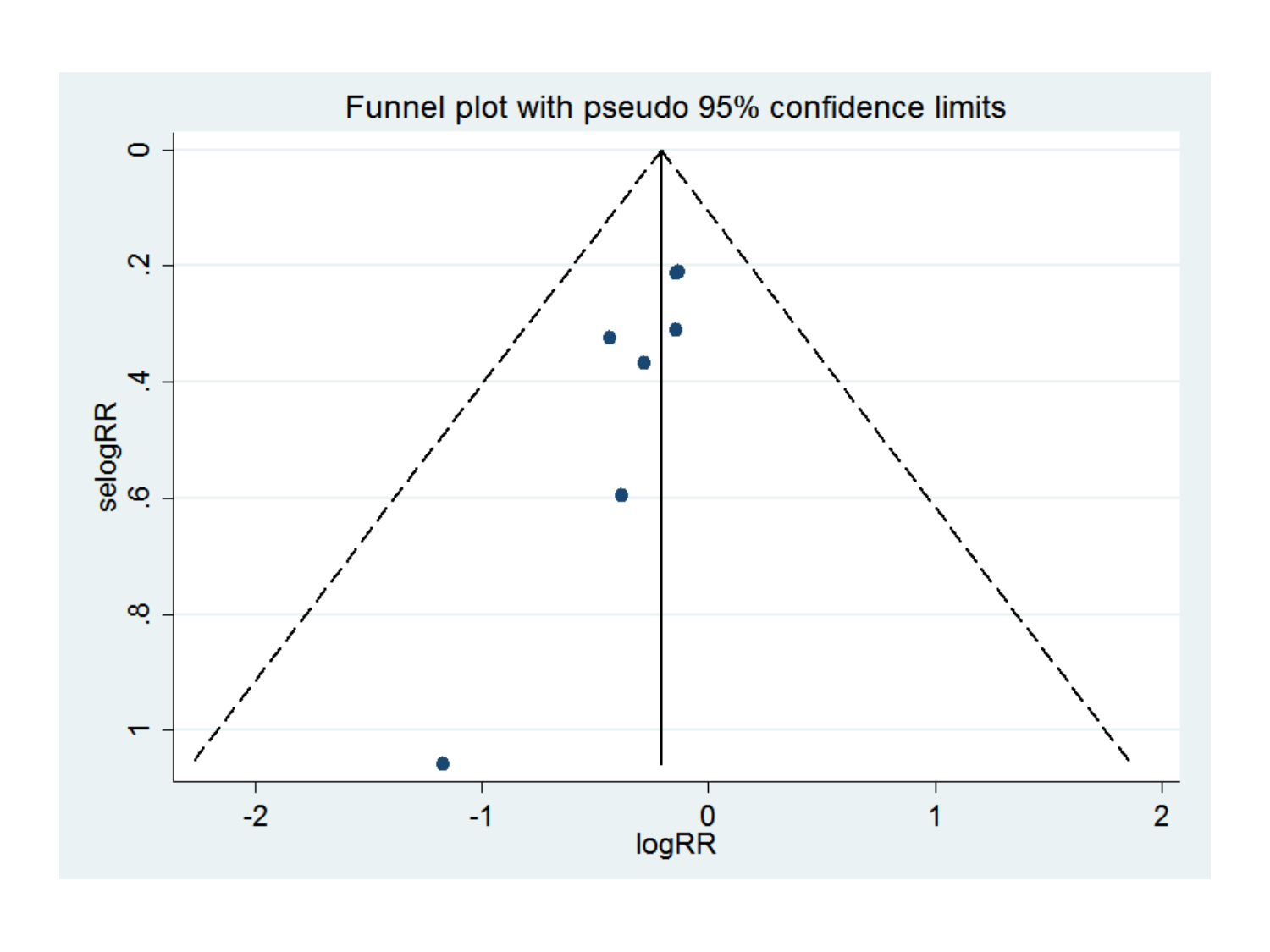

#

Supplement: Additional file 7 — Funnel plot of the association between obesity and pneumonia mortality risk. [file 1741-7015-12-61-S7.pptx]

## Slide 1
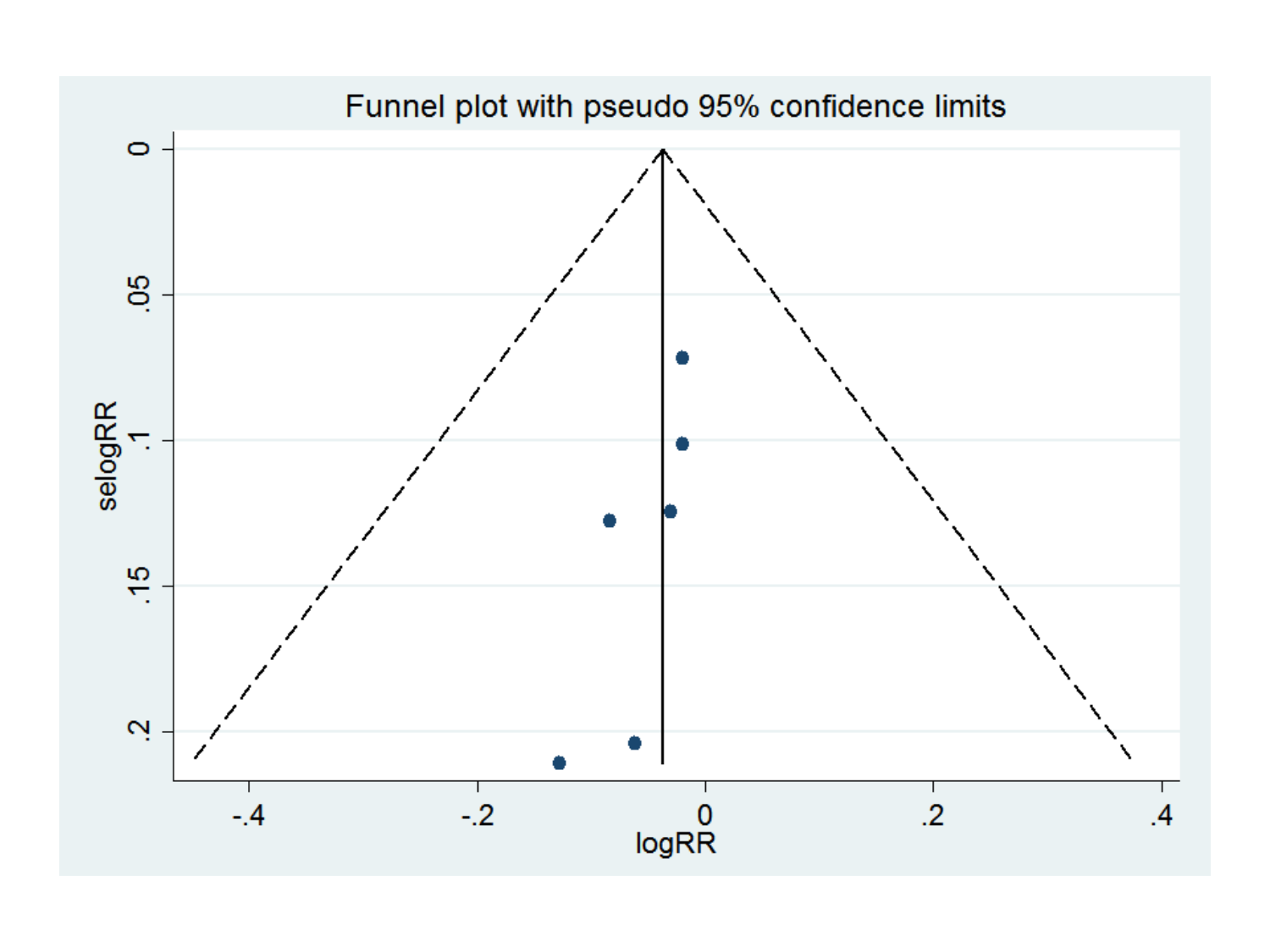

#

Supplement: Additional file 8 — Funnel plot of the association between obesity and pneumonia mortality risk in dose-response analysis. [file 1741-7015-12-61-S8.pptx]
