# Supplementary material for: Obesity survival paradox in pneumonia: a meta-analysis
Source: BMC Med. 2014 Apr 10;12:61. doi: 10.1186/1741-7015-12-61 (PMC4021571; doi:10.1186/1741-7015-12-61)
Supplement: Additional file 6 — Subgroup analyses of pneumonia risk, overweight and obesity versus normal weight and dose–response analyses, respectively. [file 1741-7015-12-61-S6.pdf]

Table S3. Subgroup analyses of pneumonia risk, overweight and obesity vs. normal weight and dose-response analyses, respectively.

	Overweight and obesity vs. normal weight						Dose-response analyses					
Subgroups	No. of studies	Relative risk (95% CI)	*P* for test	I^2 (%)	*P* for heterogeneity	*P* for meta-regression	No. of studies	Relative risk (95% CI)	*P* for test	I^2 (%)	*P* for heterogeneity	*P* for meta-regression
Study design						0.55						0.64
Prospective	7	1.28 (1.07-1.53)	<0.01	60	0.02		6	1.05 (1.02-1.07)	<0.01	52	0.06	
Retrospective	5	1.53 (0.87-2.66)	0.14	87	<0.01		4	1.04 (0.97-1.12)	0.25	86	<0.01	
Gender						0.42						0.45
Male	4	1.10 (0.73-1.64)	0.66	89	<0.01		4	1.02 (0.97-1.08)	0.47	92	<0.01	
Female	4	1.21 (0.82-1.78)	0.36	84	<0.01		4	1.04 (0.97-1.11)	0.26	80	<0.01	
Mixed	8	1.47 (1.08-2.01)	0.01	71	<0.01		6	1.05 (1.00-1.09)	0.04	68	<0.01	
Assessment of case						0.56						0.84
Physician-diagnosed	6	1.49 (1.03-2.17)	0.04	79	<0.01		4	1.05 (1.00-1.11)	0.05	82	<0.01	
ICD	3	1.30 (1.01-1.68)	0.04	63	0.04		4	1.05 (1.02-1.07)	<0.01	37	0.19	
Pneumonia type						0.69						0.50
CAP	8	1.42 (1.02-1.97)	0.04	92	<0.01		6	1.04 (1.01-1.09)	0.02	90	<0.01	
HAP	4	1.15 (0.85-1.55)	0.37	49	0.12		2	1.02 (0.97-1.07)	0.42	68	0.04	
Assessment of weight and height						0.02						0.04
Measured	8	1.15 (1.01-1.33)	0.04	25	0.23		7	1.04 (1.01-1.06)	<0.01	41	0.13	
Self-reported	2	1.80 (1.28-2.52)	<0.01	17	0.17		2	1.10 (1.06-1.13)	<0.01	0	0.32	
Duration of follow-up												0.61
>5 years	6	1.22 (0.86-1.73)	0.26	93	<0.01	0.17	6	1.04 (0.99-1.09)	0.09	90	<0.01	
≤5 years	5	1.45 (1.14-1.86)	<0.01	46	0.12		4	1.07 (1.03-1.11)	<0.01	53	0.09	

ICD, International Classification of Diseases; CAP, community-acquired pneumonia; HAP, hospital-acquired pneumonia.
