# Supplementary material for: Obesity survival paradox in pneumonia: a meta-analysis
Source: BMC Med. 2014 Apr 10;12:61. doi: 10.1186/1741-7015-12-61 (PMC4021571; doi:10.1186/1741-7015-12-61)
Supplement: Additional file 9 — Subgroup analyses of pneumonia mortality risk, overweight and obesity versus normal weight and dose–response analyses, respectively. [file 1741-7015-12-61-S9.pdf]

Table S4. Subgroup analyses of pneumonia mortality risk, overweight and obesity vs. normal weight and dose-response analyses, respectively.

	Overweight and obesity vs. normal weight						Dose-response analyses					
Subgroups	No. of studies	Relative risk (95% CI)	*P* for test	I^2 (%)	*P* for heterogeneity	*P* for meta-regression	No. of studies	Relative risk (95% CI)	*P* for test	I^2 (%)	*P* for heterogeneity	*P* for meta-regression
Study design						0.02						0.67
Prospective	5	0.75 (0.64-0.88)	<0.01	18	0.30		4	0.95 (0.92-0.99)	<0.01	75	<0.01	
Retrospective	2	0.87 (0.83-0.93)	<0.01	0	0.85		2	0.98 (0.96-0.99)	<0.01	30	0.23	
Gender						0.20						0.35
Male	2	0.66 (0.55-0.81)	<0.01	0	0.37		2	0.92 (0.89-0.95)	<0.01	0	0.34	
Female	2	0.87 (0.57-1.33)	0.53	77	0.04		2	0.97 (0.90-1.04)	0.40	70	0.07	
Mixed	5	0.86 (0.78-0.94)	<0.01	27	0.23		4	0.97 (0.94-0.99)	0.02	56	0.08	
Assessment of case						0.81						0.84
Physician-diagnosed	2	0.87 (0.80-0.95)	<0.01	0	0.58		3	0.94 (0.88-1.00)	0.04	68	0.05	
ICD	4	0.80 (0.68-0.94)	<0.01	60	0.06		3	0.96 (0.92-1.00)	0.05	87	<0.01	
Assessment of weight and height						---						---
Measured	7	0.83 (0.77-0.91)	<0.01	34	0.17		6	0.95 (0.93-0.98)	<0.01	77	<0.01	
Duration of follow-up						0.50						0.43
>5 years	4	0.84 (0.76-0.92)	<0.01	60	0.06		4	0.95 (0.91-0.99)	<0.01	85	<0.01	
≤5 years	2	0.64 (0.33-1.24)	0.18	0	0.51		1	0.94 (0.86-1.02)	0.16	---	---	

ICD, International Classification of Diseases; CAP, community-acquired pneumonia; HAP, hospital-acquired pneumonia.
